# The impact of COVID-19 on medication reviews in English primary care. An OpenSAFELY-TPP analysis of 20 million adult electronic health records

**DOI:** 10.1111/bcp.16030

**Published:** 2024-03-26

**Authors:** The OpenSAFELY Collaborative, C Wood, V Speed, L Fisher, HJ Curtis, AL Schaffer, AJ Walker, R Croker, AD Brown, C Cunningham, WJ Hulme, CD Andrews, BFC Butler-Cole, D Evans, P Inglesby, I Dillingham, SCJ Bacon, S Davy, T Ward, G Hickman, L Bridges, T O’Dwyer, S Maude, RM Smith, A Mehrkar, C Bates, J Cockburn, J Parry, F Hester, S Harper, B Goldacre, B MacKenna

**Affiliations:** 1 Bennett Institute for Applied Data Science, Nuffield Department of Primary Care Health Sciences, University of Oxford, OX2 6GG, UK; 2 Department of Thrombosis and Haemostasis, King’s College Hospital, Denmark Hill, London, SE5 9RS; 3 TPP, TPP House, 129 Low Lane, Horsforth, Leeds, LS18 5PX, UK

**Keywords:** Primary care, General practice, Prescribing

## Abstract

**Aims:**

The COVID-19 pandemic caused significant disruption to routine activity in primary care. Medication reviews are an important primary care activity ensuring safety and appropriateness of prescribing. A disruption could have significant negative implications for patient care. Using routinely collected data, our aim was first to describe codes used to record medication review activity and then to report the impact of COVID-19 on the rates of medication reviews.

**Methods:**

With the approval of NHS England, we conducted a cohort study of 20 million adult patient records in general practice, in-situ using the OpenSAFELY platform. For each month, between April 2019-March 2022, we report the percentage of patients with a medication review coded monthly and in the previous 12 months with breakdowns by regional, clinical and demographic subgroups and those prescribed high-risk medications.

**Results:**

In April 2019, 32.3% of patients had a medication review coded in the previous 12 months. During the first COVID-19 lockdown, monthly activity decreased (-21.1% April 2020), but the 12-month rate was not substantially impacted (-10.5% March 2021). The rate of structured medication review in the last 12 months reached 2.9% by March 2022, with higher percentages in high-risk groups (care home residents 34.1%, 90+ years 13.1%, high-risk medications 10.2%). The most used medication review code was *Medication review done 314530002* (59.5%).

**Conclusions:**

There was a substantial reduction in the monthly rate of medication reviews during the pandemic but rates recovered by the end of the study period. Structured medication reviews were prioritised for high-risk patients.

## Background

The COVID-19 pandemic has significantly affected the capacity and delivery of services within the National Health Service (NHS).^1,2^ Many routine tasks in general practice, such as laboratory testing and blood pressure checks, were severely impacted by the COVID-19 pandemic.^3–5^

Medication reviews are a frequently undertaken task in primary care. The National Institute for Health and Care Excellence (NICE) define a medication review as ‘a structured, critical examination of a patient’s medicines with the objective of reaching an agreement with the patient about treatment, optimising the impact of medicines, minimising the number of medication related problems and reducing waste.’^6^ Medication reviews in primary care range in clinical complexity, duration and health care resource utilisation. They can be undertaken by a range of health care professionals including general practitioners, pharmacists, and nurse practitioners. There is no current national specification for target groups or frequency of medication review, however it is generally accepted that all patients who are on medications for long-term conditions should have an annual review as a minimum.^7^

In a recent UK study of 591,726 people aged over 65 and who were prescribed at least one medication, approximately half had a recorded medication review in 2019. Of those that had a medication review recorded, most were prescribed cardiovascular medications (79.9%) and most often had a diagnosis of hypertension (48.8%) or dyslipidemia (56.5%).^8^ Evidence supporting the clinical and cost effectiveness of medication reviews are mixed.^6^ A systematic review on the impact of pharmacist-led medication reviews in the community setting showed a positive impact on clinical markers for hypertension, diabetes and cholesterol. However, there were conflicting results for hospitalisations, and no reduction in mortality.^9^

The activity of undertaking a medication review and any associated actions is recorded in the electronic health record (EHR) either through manual entry of relevant clinical codes or via a built-in function (such as a template or a medication review button that appears on the repeat prescription landing page within the TPP EHR). Centralised data from EHRs can be used to study medication review activity in primary care. However, this is complicated by the array of codes used to record medication reviews, and the quality of clinical coding in practice.^10,11^

A new medication review service was launched by NHS England in September 2020.^12^ The new service focuses on offering a structured medication review (SMR) to patients at greatest risk of harm from their medications. SMRs are a patient centred, evidence-based review of a patient’s medications, taking into consideration efficacy and safety, underpinned by shared decision making. Unlike a routine medication review which may be for a *single item or therapeutic area*, an SMR is a formalised comprehensive assessment of *all medicines* a patient might be taking, with consideration of all aspects of a patient’s health. In a National Overprescribing Review Report for England, a project was highlighted that found that pharmacists undertaking SMRs were able to safely reduce prescribing by 17.4% through cessation of medicines no longer indicated or that were causing harm.^13,14^ The new SMR service is led at a practice level by pharmacists with the support of the multidisciplinary team and should target specified priority groups.^12^ The SMR service was launched during a challenging period in primary care with competing pressures such as the roll out of the first COVID-19 vaccinations, and with some lockdown restrictions still in place.

OpenSAFELY is a new secure analytics platform for electronic patient records built by our group on behalf of NHS England to deliver urgent academic and operational research during the pandemic.^15–17^ Analyses can currently run across all patients’ full raw pseudonymised primary care records at 40% of English general practices where TPP EHR software is deployed (OpenSAFELY-TPP), with patient-level linkage to various sources of secondary care data.

We set out to describe the impact of COVID-19 on all medication review activity within primary care in England using OpenSAFELY-TPP. First, we describe the individual code usage for medication reviews and then the frequency and variation of medication review activity during the COVID-19 pandemic across important demographic, regional and clinical subgroups including patients prescribed high-risk medications. Finally, we describe the launch of the SMR service in terms of frequency and variation according to the same demographic, regional, and clinical subgroups.

## Methods

### Data Source

All data were linked, stored and analysed securely within the OpenSAFELY platform: https://opensafely.org. Data include pseudonymised data such as coded diagnoses, medications and physiological parameters. No free text data are included. All code is shared openly for review and re-use under MIT open license https://github.com/opensafely/medication-reviews. Detailed pseudonymised patient data are potentially re-identifiable and therefore not shared.

### Study Design

General practice clinical activity was described by conducting a retrospective cohort study using patient-level data from English NHS general practices.

### Study Population

All patients that were alive, had a recorded age between 18 - 120 and were registered with any practice using TPP EHR software were included at each timepoint. Demographic, regional and clinical data were collated based on coded events reported between April 2019 and March 2022. Coded events may be entered manually by practice staff or generated automatically when certain activities are carried out such as completing templates, or derived from external sources such as laboratory test results.

### Codelist development

Our codelists were based on the SNOMED CT structured clinical vocabulary, which is a required standard across the NHS. We developed a “medication review” codelist^18^ using the parent terms *Review of medication 182836005* and *Medication review done 314530002* and all corresponding child codes. All codes were reviewed by two pharmacists (VS & CW) to ensure appropriateness. The codelist is openly available for inspection and re-use.^18^ We used the nationally mandated code *Structured medication review 1239511000000100* to identify SMRs.^19^

To compare usage for medication review codes, usage of each code was summarised as total counts across the study period.

### Demographic, regional and clinical subgroups

We included the following demographic categories: sex, age, Index of Multiple Deprivation (IMD) quintiles, region of registered practice, and ethnicity at 6-level and 16-level breakdowns. Ethnicity data were reported using primary care coding based on an existing codelist^20^, or where this was not present, using ethnicity data from the hospital admission data.^21^ Patients were also categorised into those with and without a primary care record of learning disability^22^, and/or of living at a nursing/care home.^23^

### Practice level variation

Practice level data are presented as decile charts, where practice level rates are extracted, ranked each month and then deciles of activity calculated. The median and interdecile range (IDR), which is the difference between the first and the ninth deciles, are compared at the time points described above.

### High-risk medications

We selected high-risk medications based on recommendations by NHS England (NHS leadership body), the Care Quality Commission (English healthcare regulator), the Medicines & Healthcare products Regulatory Agency (medicines, medical devices and blood components for transfusion regulator in the UK) and expert clinical groups.^12,24–26^ We pragmatically selected our subgroups as i) Potentially addictive medicines (benzodiazepines, ‘Z-drugs’, gabapentinoids and high dose long acting opioids) ii) Disease-modifying anti-rheumatic drugs (DMARDs) and iii) Teratogenic medicines prescribed in women of childbearing age (For the purpose of these analyses, defined as women ≤55 years).^27^ Patients were reported as prescribed a high-risk medication if they had received two or more issues of medication(s) within a subgroup in the previous 12 months. Medication codelists were derived from pseudo British National Formulary (BNF) codes that were then converted to NHS dictionary of medicines and devices (dm+d) codes and are available at OpenSAFELY Codelists:^28,29,30^ Medications included in these codelists are summarised in Supplementary Table S1.

### Study Measures

We developed measures of medication reviews carried out monthly and in the previous 12 months. Each measure was calculated as a percentage where the numerator was the cohort of patients with a coded medication review either within that month or within the previous 12 months depending on the measure, and the denominator was all patients in the selected study population within that time period. Time-periods were referred to as single months, where a single month captures all events occurring up to and including the last day of a reported month. For the 12-month measure, each month includes activity occurring within the reported month or previous 11 months.

Where multiple codes from a single codelist were recorded in the patient record in a single month only the latest record was returned to calculate the measure. The measures described above were repeated for SMRs alone as a separate analysis.

### Classification of change

The rate of monthly medication reviews and the rate of medication reviews in the previous 12 months was compared to April 2019 which we defined as the “baseline”. The change from baseline was classified according to Box 1, using previously developed methods, based on percentage change.^3,5^

### Statistical methods

The percentage of patients having medication reviews was standardised by both age (5-year age bands) and sex using the Office for National Statistics (ONS) mid-year 2020 English population^31^ for comparison between relevant demographics (ethnicity, IMD quintile, region, age and sex). The change in the percentage of patients that had had a medication review in the previous 12 months and those who had not was compared between baseline (April 2019) and March 2021 (12 months after the initial lockdown restrictions were implemented) and March 2022 (the final month of these analyses).

To minimise disclosivity, small counts (less than or equal to 7) were suppressed, final counts were then rounded to nearest five. True zero values were retained for the medication review code usage.

### Software and Reproducibility

Data management and analysis was performed using Python 3.8. Code for data management and analysis as well as codelists is openly available for inspection and re-use at https://github.com/opensafely/medication-reviews.

### Patient and Public Involvement

We have developed a publicly available website https://opensafely.org/ which describes the platform in language suitable for a lay audience. We have participated in two citizen juries exploring trust in OpenSAFELY.^32^ On our OpenSAFELY Oversight Board we have patient representation and are currently co-developing an explainer video for our platform. We have also partnered with Understanding Patient Data to produce lay explainers on the importance of large datasets for research and regularly participate in online public engagement events to important communities (for example, Healthcare Excellence Through Technology; Faculty of Clinical Informatics annual conference; NHS Assembly; and the Health Data Research UK symposium. Further, we are working closely with appropriate medical research charities, for example, Association of Medical Research Charities, to ensure the patient voice is reflected in our work. We share the interpretation of our findings through press releases, social media channels, and plain language summaries.

## Results

At baseline in April 2019 the monthly percentage of patients with a medication review coded in April 2019 was 3.8%, substantially decreasing in the first COVID-19 lockdown (April 2020) period to 3.0% (-21.1% from baseline) but by March 2022 recovering to 4.0% (+5.3% from baseline).

In April 2019 the percentage of patients who had a medication review coded in the previous 12 months was 32.3% (6,249,415/19,357,210). By March 2021, this figure reduced to 28.9% (5,725,135/19,856,170), reflecting a 10.5% decrease compared to the initial baseline, classified as no substantial change. In March 2022, the most recently reported percentage of patients with a medication review in the previous 12 months was 29.6% (5,977,300/20,181,035) an 8.4% reduction from baseline, classified as no substantial change.

Demographic, regional, and clinical characteristics of the study population are reported in Table 1 according to the final month of the study period (March 2022). The percentage of patients with medication reviews monthly and in the previous 12 months, are shown in Supplementary Figure 1.

## Codelist analysis

Table 2 details the top 10 medication review codes used across the study period. *Medication review done 314530002* was most frequently used to report medication review activity (59.5%), with all other codes individually accounting for <5% of activity.

### Demographic, regional and clinical subgroups

**Figure 1:**
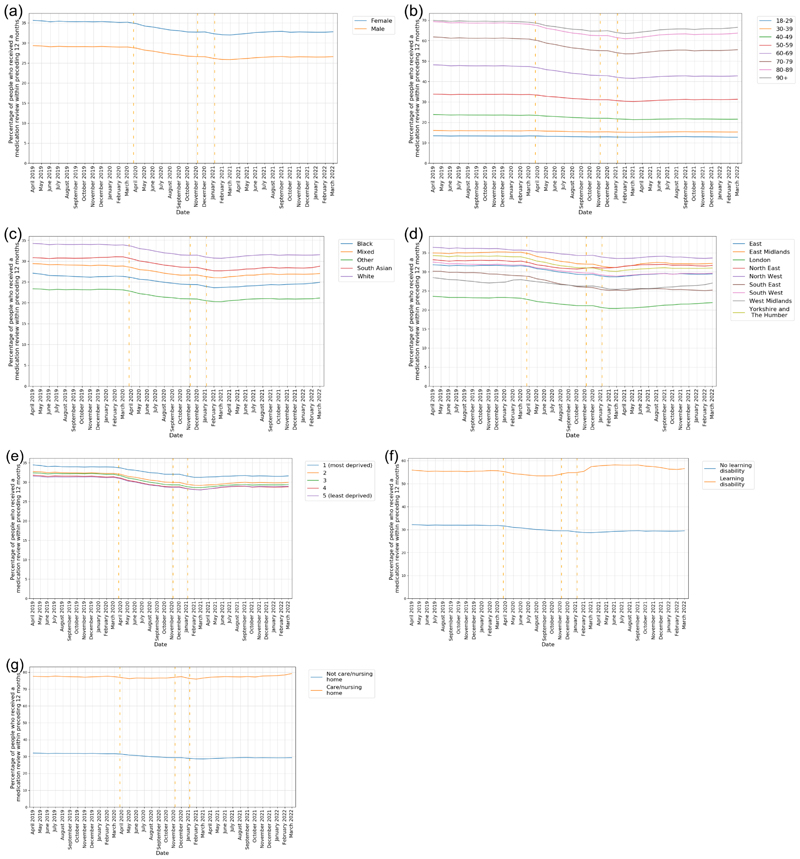
The percentage of patients that had had a medication review in the previous 12 months, reported monthly for the period April 2019 to March 2022 (inclusive) stratified by a) Sex (age standardised) b) Age bands (sex standardised) c) Ethnicity (age/sex standardised) d) Region (age/sex standardised) e) IMD quintiles (age/sex standardised) f) Record of learning disability g) Record of living in a nursing/care home. Vertical dashed lines represent the start of three lockdown periods (23rd March 2020, 5th November 2020, 5th January 2021).

Female patients consistently had a higher rate of medication review completed within the previous 12 months than male patients when adjusted for age (32.8% vs 26.6%, March 2022) (Figure 1a).

Advancing age was associated with an increasing percentage of patients having received a medication review in the previous 12 months (Figure 1b). In March 2022, for patients aged 70-79, 55.6% had a medication review in the previous 12 months, increasing to 66.5% in patients aged over 90 years.

After age-sex standardisation there remains underlying variation in the percentage of patients with a medication review in the previous 12 months according to ethnicity and region (Figure 1c & 1d). Patients with Other and Black ethnicity and those living in London, the South-East and the West Midlands have consistently lower percentages of medication reviews in the previous 12 months. Notably, we observed a trend for recovery in the West Midlands but a decline in the South East after the end of the COVID-19 restrictions.

When stratified by IMD, the crude rates show the lowest rate of reviews in the previous 12 months occur in the most deprived areas but after age/sex standardisation this is reversed with the highest rate amongst those living in the most deprived areas (Figure 1e).

During the pandemic, there was a decrease in the percentage of reviews for patients with a record of learning difficulties or in nursing or care homes per month, but activity resumed relatively quickly (Figure 1f & 1g).

Breakdowns of the percentage of patients with medication reviews in the previous 12 months, according to all demographic, regional and clinical breakdowns at baseline, March 2021, and March 2022 are reported in the Supplementary Table 2.

### Practice level variation

**Figure 2:**
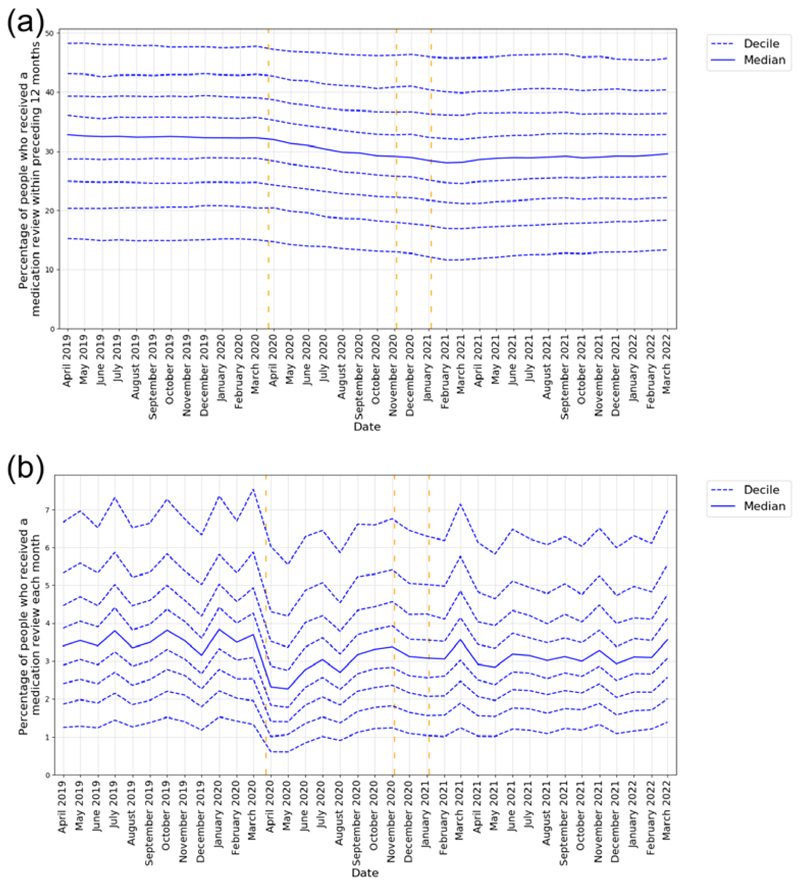
Practice level decile plots of medication review activity for the period April 2019 to March 2022 (inclusive): Percentage of patients with: a) Medication review recorded in the previous 12 months b) Medication review recorded monthly. The median percentage is displayed as a thick blue line and deciles are indicated by dashed blue lines. Vertical dashed lines represent the start of three lockdown periods (23rd March 2020, 5th November 2020, 5th January 2021). All deciles are calculated across 2546 OpenSAFELY-TPP practices.

Practice level decile plots, which show variation between practices, are reported in Figure 2. The practice median of patients with a medication review coded in the previous 12 months closely followed the overall trend (April 2019 32.8%, March 2021 28.1%, March 2022 29.6%), the IDR increased slightly during the pandemic but recovered by the end of the study period (April 2019 1^st^ decile 15.3%, 9^th^ decile 48.3%, IDR 33.0%, March 2021 1^st^ decile 11.6%, 9^th^ decile 45.8%, IDR 34.2%, March 2022 1^st^ decile 13.3%, 9^th^ decile 45.8%, IDR 32.5%).

### High-risk medications

**Figure 3:**
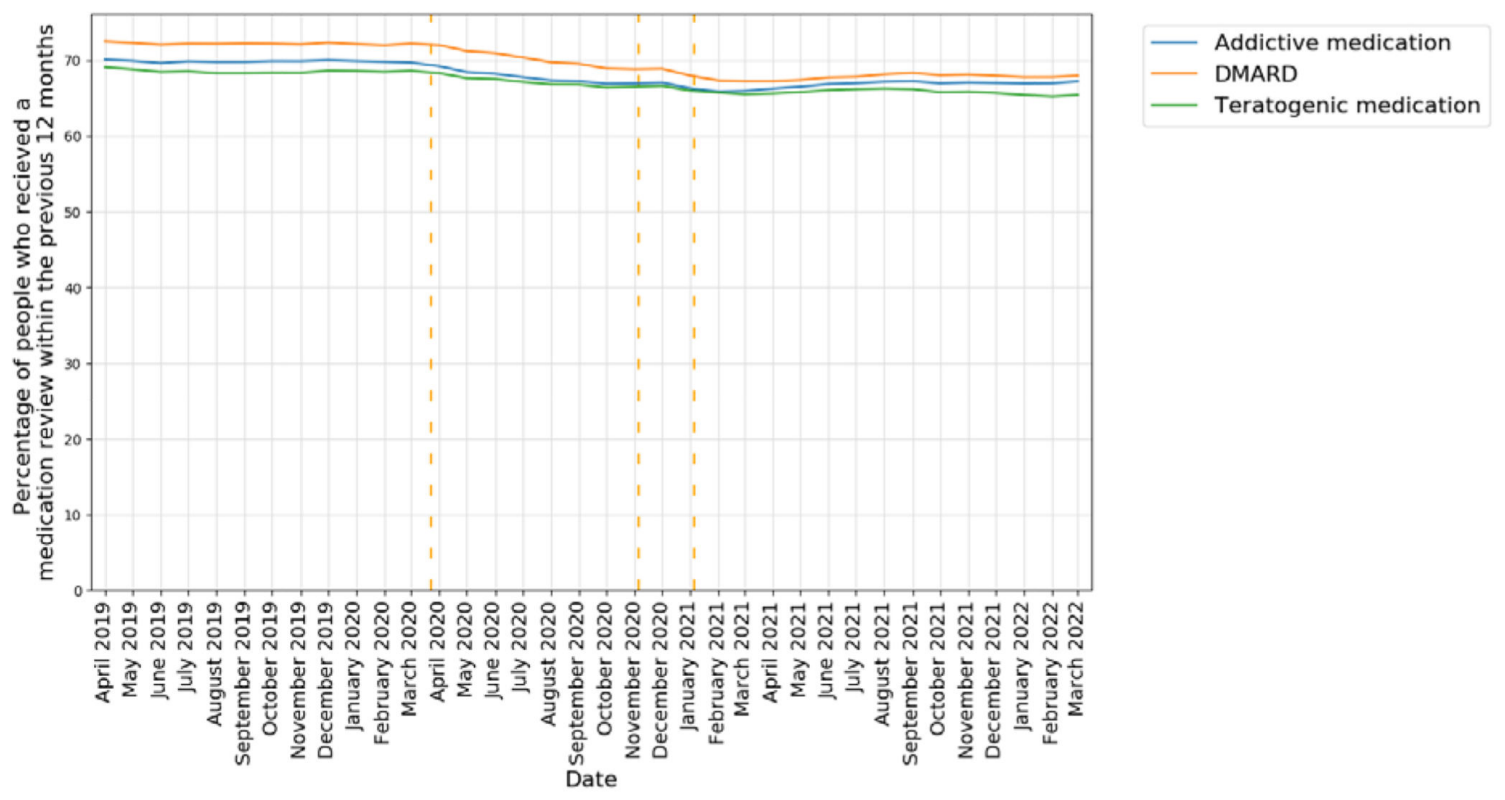
The percentage of patients with two or more prescriptions in the previous 12 months for a high-risk drug that had had a medication review in the previous 12 months, reported monthly for the period April 2019 to March 2022 (inclusive). Vertical dashed lines represent the start of three lockdown periods (23rd March 2020, 5th November 2020, 5th January 2021).

The percentages of patients prescribed a high-risk medication who had a record of a medication review in the previous 12 months, are reported in Figure 3. In April 2019, 70.1% of patients prescribed a potentially addictive medication had a record of a medication review in the previous 12 months, this reduced to 66.0% in March 2021 (-5.8%), and then showed some improvement, increasing to 67.2% in March 2022 (-4.1%). At baseline, 72.5% of patients prescribed a DMARD had had a medication review in the previous 12 months, this reduced to 67.2% in March 2021 (-7.3%), and remained largely unchanged at 68.0% in March 2022 (-6.2%). For female patients of childbearing age prescribed a potentially teratogenic medicine, 69.1% had had a medication review in the previous 12 months at baseline. This reduced to 65.5% in March 2021 (-5.2%) and remained unchanged 65.4% in March 2022 (-5.4%).

### Structured medication reviews

Following the launch of the SMR service in September 2020, the percentage of patients having an SMR recorded within the previous 12 months increased to 2.9% by March 2022. The rate of increase reduced from September 2021 onwards, 12 months after the release of SMR guidance.

**Figure 4:**
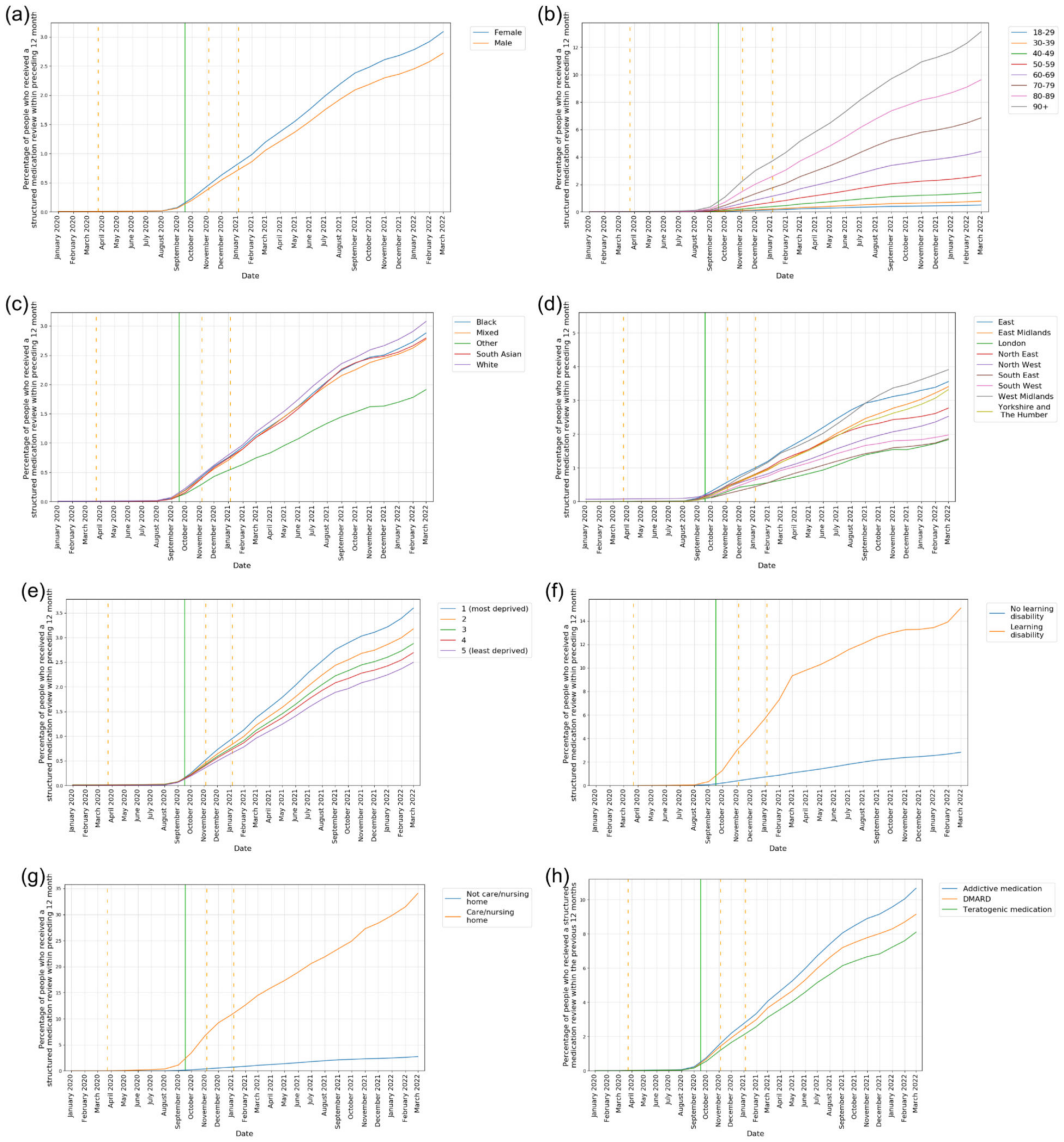
The percentage of patients that had had a structured medication review in the previous 12 months, reported monthly for the period January 2020 to March 2022 (inclusive) stratified by a) Sex (age standardised) b) Age bands (sex standardised) c) Ethnicity (age/sex standardised) d) Region (age/sex standardised) e) IMD quintiles (age/sex standardised) f) Record of learning disability g) Record of living in a nursing/care home h) High-risk medications. Vertical orange dashed lines represent the start of three lockdown periods (23rd March 2020, 5th November 2020, 5th January 2021). Vertical green line represents the launch of Structured Medication Review guidance (17th September 2020).

In keeping with the results for all medication reviews, female patients and those of advancing age consistently had a higher percentage of SMRs recorded within the previous 12 months (female 3.1% vs male 2.7% (adjusted for age)) and (90+ years 13.1%, 80-89 years 9.6%, 70-79 years 6.9% (adjusted for sex)) respectively in March 2022 (Figure 4a & 4b).

After age-sex standardisation there remains underlying variation according to ethnicity and region (Figure 4c & 4d). Patients with Other ethnicity and those living in London, the South East and the West Midlands had consistently lower percentages of patients with an SMR recorded in the previous 12 months.

When stratified by IMD, the highest percentage of SMRs recorded in the previous 12 months was amongst those living in the most deprived areas (Figure 4e). Patients with a record of learning difficulties or with a record of living in a nursing or care home had substantially higher percentages of SMRs recorded in the previous 12 months (15.1%, 34.1%, respectively) (Figure 4f & 4g).

By March 2022, patients prescribed high-risk medications had a higher percentage of SMRs completed within the previous 12 months (10.2%) than the study population overall. Those prescribed potentially addictive medication showed the highest percentage (10.7%), followed by those prescribed DMARDs (9.1%) and then female patients of childbearing age prescribed a potentially teratogenic medicine (8.1%) (Figure 4h).

## Discussion

This study reports the rate of medication reviews during the COVID-19 pandemic in approximately 20 million patients. During the COVID-19 pandemic there was a substantial decrease in the rate of medication reviews taking place in England per month. However, the percentage of patients having a medication review coded in the previous 12 months was less impacted with a much smaller reduction (-10.5%), indicating a rapid recovery within primary care. During a period of stretched resources and national lockdown restrictions, our results demonstrate prioritisation of workload, with older patients, patients in care homes, patients with learning difficulties and those prescribed high-risk medications receiving a higher frequency of medication reviews. This study also demonstrates rapid deployment of a national SMR service in September 2020 and those at greatest risk were prioritised.^12^

### Comparison with existing literature

The COVID-19 pandemic has had a substantial impact on the delivery of healthcare worldwide since its first peak in early 2020. In December 2021, The World Health Organisation shared a third report on the continuity of essential health services. 117/127 (92%) countries continued to experience disruption in at least one essential health service, with 53% reporting ongoing disruption in primary care.^34^ Consistent with these data, and OpenSAFELY NHS Service Restoration Observatory studies^3–5^, we observed disruption in the delivery of medication reviews during the pandemic.

In this manuscript, we expand on our previous work which described the frequency of medication reviews in England during the pandemic.^4,5^ Our results align with this previous work showing *Medication review done* (314530002) represented the major code used within TPP EHR. We have previously demonstrated that the choice of EHR system may influence prescribing and coding activity.^5,35–37^ In a study reporting general practice activity we found substantial differences in the medication review codes used in TPP and EMIS EHRs.

We also report for the first time, regional, demographic, and clinical variation in recorded medication reviews amongst 20 million patients in primary care.^38,39^ The next largest study, a recent report using UK Clinical Practice Research Datalink reported living in a care home, baseline prescription count, and having a medication review in the previous year as the strongest predictors of having a medication review in 2019. Consistent with the findings of this study, the investigators observed geographical variation in the frequency of medication review but no substantial variation according to deprivation. However, they were unable to meaningfully evaluate the influence of ethnicity due to missing data.^8^

Structured medication review appointment counts are publicly available from August 2021, based upon NHS digital appointment data, categorised by context type and region. In March 2022, 195,229 appointments categorised as SMRs took place across all patients in England.^40^ This compares with 77,295 SMR codes recorded in the same month in our analysis (39.6% of total), in keeping with the 40% coverage of the English population with OpenSAFELY-TPP. A qualitative study reporting semi-structured interviews with pharmacists in primary care described uncertainty in the identification and prioritisation of patients for SMR.^41^ We report a favourable picture of the prioritisation of medication reviews in patients potentially at a greater risk of harm from medicines.

### Implications for research and/or practice

Coding medication reviews is complex. First, there are a high number of codes that relate to medication review activity in primary care with no guidance or national audit to determine which codes are preferred, with the exception of SMRs for which a single code is used. Medication review code descriptions are typically broad and more specific terms are not frequently used unless there is a requirement to demonstrate activity elsewhere (for example, the Asthma medication review code (394720003) belongs to a cluster of codes used in the Quality and Outcomes Framework (QOF) for asthma^42^). The most frequently used medication review codes reported here are consistent with those listed in the NHS Digital Primary Care Domain Refset for medication reviews^38^ and the now deprecated Care Planning Medication Review Refset^39^. To enable more consistent and meaningful data on medication reviews we recommend that there be a national review of medication review codes to i) curate a reference set including a small number of preferred medication review codes ii) provide and support the regular review of metadata that describes important limitations or considerations for medication review coding iii) provide guidance to EHR providers regarding the preferred codes/picking lists for medication review activity.

The OpenSAFELY platform is a valuable tool for national organisations such as NHSE, CQC and MHRA to monitor adherence to national guidelines and variation in practice. In this study, we have demonstrated that there is variation in the percentage of patients having a medication review according to region and ethnicity. We recommend that national bodies use OpenSAFELY to identify and target these differences to improve the quality of care, particularly in patients at risk of health inequalities. The OpenSAFELY collaborative is constructing the Core20PLUS5 (a national NHS England approach to reducing healthcare inequalities) as code for re-use by OpenSAFELY users.^43,44^

### Strengths and limitations

Using the OpenSAFELY platform we are able to report completion of routine tasks in primary care such as medication reviews at scale. In this study, we used routinely collected data from 20 million patient records from practices using TPP EHR. In general TPP registered patients have been found to be generally representative of the English population as a whole in terms of key demographic characteristics.^33^ Through OpenSAFELY, patient-level data is securely linked to enable analyses to identify important demographic, clinical and regional variation.

An important limitation of this analysis, we have not identified patients who are on regular repeat medications which could help establish individuals’ need for a medication review. We are rapidly developing the OpenSAFELY platform and we will add this functionality to support future studies. In this study we pragmatically identified selected groups who would most likely benefit from a medication review such as those prescribed high-risk medications. We did not correct for variation in patient needs between practices which could explain reasonable variation between practices. Accuracy of clinical coding is a limitation of all EHR research into clinical conditions and activity^11^ and our approach relies on a clinician adding an appropriate clinical code to indicate a medication review has been done. Our summary of medication review code usage demonstrates the range of SNOMED CT codes used in clinical practice to report the same activity. To overcome uncertainty about the codes selected in practice, we took an inclusive approach to ensure that we captured all activity relating to medication reviews. For future research, we have shared detailed code usage for medication reviews (Supplementary Table 3).

## Conclusion

There was a substantial decrease in the rate of medication reviews taking place in England per month during the COVID-19 pandemic. However, the percentage of patients having a medication review coded in the previous 12 months was less impacted, indicating a rapid recovery within primary care. The national SMR service was rapidly deployed after launch, with those at greatest risk being prioritised.

## Supplementary Material

Supplementary Material

## Figures and Tables

**Table 1:** Patient characteristics and rates of medication reviews in the study population (Registered adult patients ≥18 years) in the previous 12 months (March 2022)

	No. registered patients		No. with medication review	Percentage	
	n	% of total	n	Crude	Age and sex standardised
**Total**	20,181,035	100	5,977,300	29.6	29.7
**Sex**					
Female	10,130,280	50.2	3,382,280	33.4	32.8
Male	10,050,750	49.8	2,595,020	25.8	26.6
**Age**					
18-29	3,671,600	18.2	461,525	12.6	12.7
30-39	3,618,395	17.9	545,490	15.1	15.3
40-49	3,235,545	16.0	688,570	21.3	21.5
50-59	3,444,720	17.1	1,073,915	31.2	31.3
60-69	2,755,735	13.7	1,179,060	42.8	42.8
70-79	2,207,055	10.9	1,226,540	55.6	55.6
80-89	1,023,650	5.1	652,245	63.7	63.7
90+	224,335	1.1	149,955	66.8	66.5
**IMD quintile**					
1 (most deprived)	3,800,990	18.8	1,068,930	28.1	31.6
2	3,917,190	19.4	1,110,860	28.4	29.9
3	4,243,805	21.0	1,273,175	30.0	29.4
4	3,999,795	19.8	1,218,085	30.5	28.8
5 (least deprived)	3,659,350	18.1	1,153,700	31.5	29.0
Unknown	559,905	2.8	152,545	27.2	31.0
**Region**					
East	4,590,260	22.7	1,352,230	29.5	29.4
East Midlands	3,501,525	17.4	1,131,750	32.3	32.2
London	1,498,030	7.4	244,325	16.3	21.9
North East	935,195	4.6	293,685	31.4	31.6
North West	1,736,550	8.6	602,985	34.7	33.6
South East	1,340,960	6.6	348,755	26.0	25.2
South West	2,837,955	14.1	898,790	31.7	29.6
West Midlands	786,790	3.9	200,715	25.5	27.0
Yorkshire and The Humber	2,887,260	14.3	886,260	30.7	31.0
Unknown	66,510	0.3	17,810	26.8	30.0
**Ethnicity**					
British	13,685,595	67.8	4,872,060	35.6	33.0
Irish	107,435	0.5	34,175	31.8	28.0
Any other White background	2,036,095	10.1	353,195	17.3	23.4
Indian	611,885	3.0	133,690	21.8	28.5
Pakistani	411,425	2.0	93,420	22.7	31.6
Bangladeshi	97,525	0.5	21,165	21.7	31.0
Any other Asian background	346,215	1.7	60,475	17.5	25.1
African	298,835	1.5	45,495	15.2	22.7
Caribbean	111,405	0.6	31,290	28.1	27.9
Any other Black background	84,615	0.4	16,625	19.6	25.9
White and Asian	54,450	0.3	10,160	18.7	27.2
White and Black Caribbean	61,245	0.3	13,715	22.4	29.7
White and Black African	50,095	0.2	8,440	16.8	24.2
Any other mixed background	104,790	0.5	18,725	17.9	26.5
Chinese	161,855	0.8	13,860	8.6	16.8
Any other ethnic group	297,410	1.5	45,800	15.4	23.3
Unknown	1,660,165	8.2	205,005	12.3	16.3
**Record of learning disability**	119,800	0.6	67,760	56.6	-
**Record of individual living at a care/nursing home**	112,775	0.6	89,345	79.2	-
**Record of two or more prescriptions in the previous 12 months for:**					
Potentially addictive medications	913,110	4.5	613,660	67.2	-
DMARD	171,790	0.9	116,765	68.0	-
Teratogenic medication*	112,515	0.6	73,610	65.4	-
Any high-risk medication above	1,099,095	5.4	735,790	66.9	-

* Female patients ≤55 years

**Table 2:** Top 10 codes used to report medication review activity for patients registered at TPP practices between April 2019-March 2022

SNOMED CT code	n=35,939,595	%
Medication review done (314530002)	21,382,570	59.5
Review of medication (182836005)	1,651,115	4.6
Medication review with patient (88551000000109)	1,504,035	4.2
Medication review done by clinical pharmacist (1127441000000107)	1,440,845	4.0
Medication review done by pharmacist (719329004)	1,322,265	3.7
Structured medication review (1239511000000100)	1,286,160	3.6
Dispensing review of use of medicines (279681000000105)	939,180	2.6
Medication review of medical notes (93311000000106)	884,945	2.5
Asthma medication review (394720003)	844,270	2.3
Medication review without patient (391156007)	730,365	2.0

## Data Availability

Access to the underlying identifiable and potentially re-identifiable pseudonymised electronic health record data is tightly governed by various legislative and regulatory frameworks, and restricted by best practice. The data in OpenSAFELY is drawn from General Practice data across England where TPP is the data processor. TPP developers initiate an automated process to create pseudonymised records in the core OpenSAFELY database, which are copies of key structured data tables in the identifiable records. These pseudonymised records are linked onto key external data resources that have also been pseudonymised via SHA-512 one-way hashing of NHS numbers using a shared salt. Bennett Institute for Applied Data Science developers and Principle Investigators holding contracts with NHS England have access to the OpenSAFELY pseudonymised data tables as needed to develop the OpenSAFELY tools. These tools in turn enable researchers with OpenSAFELY data access agreements to write and execute code for data management and data analysis without direct access to the underlying raw pseudonymised patient data, and to review the outputs of this code. All code for the full data management pipeline—from raw data to completed results for this analysis—and for the OpenSAFELY platform as a whole is available for review at github.com/OpenSAFELY. The data management and analysis code for this paper was led by (CW and VS) and contributed to by (LF and BMK).

## What is already known about this subject

- The COVID-19 pandemic brought substantial disruption to the delivery of routine tasks in primary care.
- For the first time on this scale, our study reports the impact of COVID-19 on medication review activity, including the launch of the structured medication review service in England broken down by key demographic, social, and clinical factors.

## What this study adds

- There was a substantial reduction in the monthly rate of medication reviews during the pandemic but rates recovered quickly.
- The percentage of patients with a medication review varies according to region and ethnicity.
- Structured medication reviews were adopted rapidly and prioritised for patients at greatest risk of harm from their medicines.

## Box 1 Service change classification relative to baseline (April 2019)

**Change from baseline:**

- **no substantial change:** activity remained within 15% of the baseline level
- **substantial increase:** an increase of >15% from baseline
- **substantial decrease:** a decrease of >15% from baseline

**For March 2022:**

- **no substantial change:** no change
- **sustained drop:** sustained drop, a decrease which has not yet returned to 15% of baseline
- **recovery:** a decrease which has returned to within 15% of baseline
